# Women want male partner engagement in antenatal care services: A qualitative study of pregnant women from rural South Africa

**DOI:** 10.1371/journal.pone.0283789

**Published:** 2023-04-03

**Authors:** Carolyn M. Audet, Daniel E. Sack, Godfrey H. Ndlovu, Caroline Morkel, Jacob Harris, Ryan G. Wagner, Tshegofatso M. Seabi

**Affiliations:** 1 Vanderbilt Institute of Global Health, Vanderbilt University Medical Center, Nashville, Tennessee, United States of America; 2 Medical Research Council, Wits Rural Public Health and Health Transitions Research Unit (Agincourt), School of Public Health, Faculty of Health Sciences, University of the Witwatersrand, Johannesburg, South Africa; 3 Notre Dame University, Notre Dame, Indiana, United States of America; University of Cape Town, SOUTH AFRICA

## Abstract

**Introduction:**

Evidence strongly shows that a supportive, involved male partner facilitates maternal HIV testing during pregnancy, increases maternal antiretroviral (ART) adherence and increases HIV-free infant survival. Partner engagement in antenatal care (ANC) is influential; however, the most effective strategy to engage male partners is currently unknown. Engaging pregnant women to understand whether male partner involvement is welcome in ANC, what this involvement entails and how best to invite their partner is an important first step in determining how best to engage male partners.

**Methods:**

We interviewed 36 pregnant women receiving ANC services at a district hospital in rural Mpumalanga, South Africa to assess the strengths and weaknesses of their current relationship, the type of partner support they receive, whether they would like their male partner to be involved in their ANC, and how best to invite their male partner to their appointments. We conducted a thematic analysis of the qualitative interviews using MAXQDA software.

**Results:**

Financial, emotional, and physical support were noted as important aspects of support currently provided by male partners, with most pregnant women wanting their partners to engage in ANC services during pregnancy. Preferred engagement strategies included participation in couple-based HIV testing and counseling, regular ANC appointment attendance, and delivery room presence. Women who reported a positive relationship with her partner were more likely to prefer inviting their partner without health facility assistance, while those who reported challenges in their relationship preferred assistance through a letter or community health worker. Pregnant women perceived regular business hours (due to their partner being employed and unable to take off work) and having a partner involved in multiple relationships as barriers in getting their partner to attend ANC services.

**Discussion:**

Rural South African women, even those in unsatisfactory relationships want their male partners to attend their ANC visits and birth. To make this possible, health facilities will have to tailor male partner engagement outreach strategies to the preferences and needs of the pregnant woman.

## Introduction

A supportive, involved male partner facilitates maternal HIV testing during pregnancy, improves maternal antiretroviral therapy (ART) initiation and adherence, HIV status disclosure, HIV prevention within couples, and decreases vertical HIV transmission [1–5]. Couples HIV testing and counseling has consistently led to improved maternal and infant outcomes among pregnant women in sub-Saharan Africa (SSA) because it allows the counselor time to address issues of concern (e.g., trust), and creates a space where both partners can be educated about the necessary treatment required for the person with newly diagnosed HIV [6–13]. In South Africa, clinical services in antenatal care (ANC) and maternity wings of most public hospitals do not include partner involvement in clinical care. Male partners are not invited to attend antenatal care (ANC) services or be present for birth, regardless of the pregnant woman’s wishes.

Pregnancy can stress intimate partner relationships, resulting in relationship conflict [14] and parental stress [15]. New couples often experience a decline in relationship satisfaction in the years following childbirth, in part because they may be focusing on the child at the expense of their intimate relationship [16, 17]. A perceived decline in relationship dedication by one partner can result in decreases in personal confidence and relationship dedication by the other partner [17]. There has been considerable work in South Africa documenting how power differentials among young women and their male partners impact relationship behaviors [18, 19], but this inequality also extends to older women and can worsen during pregnancy [20]. Power disparities between couples can exacerbate gender inequalities and hinder access to HIV testing and treatment, resulting in poorer health outcomes for both mother and child [10, 21, 22].

While studies have compared HIV testing and treatment outcomes among pregnant women randomized to different strategies–verbal invitations, invitation letters from clinic, community health worker (CHW) outreach to male partners, and non-financial incentives all increase couples HIV testing and counseling during pregnancy [5, 6, 8, 13, 23–26]–little is known about how women prefer their male partners to be engaged [27]. We hypothesize that women in difficult relationships with poorer communication may prefer different outreach strategies than women in stable relationships with more supportive male partners.

Patient choice in clinical care decisions can have a positive effect on reducing loss to follow up, improving treatment retention, and clinical outcomes in specific patient populations [28, 29]. Furthermore, patient preferred treatment has been associated with improved care outcomes including lower treatment non-adherence and an increased therapeutic alliance (i.e., agreement on goals of treatment, tasks, and reciprocal positive feelings between the provider and client) [28–31]. While male partner engagement in ANC is strongly correlated with increased uptake of HIV testing and counseling, as well as treatment (if applicable), the *best strategy* for engaging a male partner in pregnancy is likely context dependent and relationship dependent, therefore, women likely know which strategy will be most effective for them and their male partner.

In this qualitative study of women attending ANC services in rural South Africa, we elicited perspectives on the strength of their relationship, if they were interested in their male partners attending ANC appointments, and which—if any—engagement strategy was preferred. We aimed to gain a deeper understanding of how to deliver personalized, patient-centered care to pregnant women in rural South Africa.

## Materials and methods

### Ethical approvals

We have approval from the Human Research Ethics Committee (Medical) at the University of the Witwatersrand (No. M200984, approved 20/11/2020) and Vanderbilt University Institutional Review Board (IRB #202035, approved 11/9/2020). The CEO of the study hospital approved our research protocol before recruitment. Consent to participate (include appropriate consent statements): Written informed consent was obtained from all participants in the study, including consent for publication.

### Data collection

We recruited pregnant women attending ANC services at Tintswalo Hospital, an acute care hospital that sees 800 women for ANC services each month– 31.1% with HIV–in Bushbuckridge, Mpumalanga province, South Africa. Pregnant, adult women (≥ 18 years old) attending ANC services were invited to participate in one in-depth interview after their ANC appointment. We approached and recruited 36 pregnant women from May 8, 2021, to June 8, 2021 using convenience sampling. None refused to participate.

We conducted interviews until we reached data saturation–initially estimated to be after 30 interviews [32]. We collaborated with local partners (authors TS and GHN) to avoid inadvertently stigmatizing participants with interview questions. Interviews initially assessed a participant’s views on the strengths and weaknesses of their relationship with their male partner, and the types of support their partner provides. We then asked participants if they would like their male partner to accompany them to their ANC appointments and, if so, how they would prefer to invite their partner. We offered three options: 1) verbally inviting their male partner; 2) a letter of invitation sent from the health facility; or 3) a community health care worker (CHW) visit for counseling and invitation. Additionally, participants were encouraged to suggest other approaches. Finally, we asked how participants would like the health facility to include their partners during pregnancy. We suggested four options: 1) couples counseling, 2) participation during ANC visits, including education from the nurse/physician, 3) couple-based HIV testing and counseling, and 4) having their male partner in the delivery room. Notes were taken during each interview. Interviews ranged from 18 to 36 minutes long.

We interviewed pregnant women receiving ANC services at a public health facility that does not currently encourage male partners to attend ANC, in part due to a lack of suitable facilities (private space) to facilitate male partner presence. While this health facility is open to change, more information from women was thought to be useful to informing future clinical practice.

Eligible participants were interviewed face-to-face in a private room with an interviewer fluent in the participant’s preferred language (generally xi-Tsonga). Interviews were recorded. Only minor children were allowed to accompany participants in the interview room. Author GHN (male) and interviewer EN (female) conducted all interviews. Neither interviewer had previously worked at the health facility nor were they acquainted with any participants. Both were employed as interviewers for the Agincourt Rural Health Unit and had experience conducting interviewers in the past. GHN identifies as a man and EN identifies as a woman. Neither had a specific interest in the topic, but both are from the community and were familiar with the difficulties women face during pregnancy. Interviews were then transcribed and translated, which GHN and EN performed independently to ensure reliable translations. Authors JH, CM, and CMA read through the transcriptions multiple times to familiarize themselves with the data and assess for data saturation.

#### Author reflexivity

The authors recognize that they have different lived experiences in their relationships than the participants. Apart from GN, the authors are not from this province of South Africa (and several of the authors from the US and Canada). Given that the authors perceptions of a “satisfactory” or “unsatisfactory” relationship were important in the development of our relationship codes, ensuring that they reflected local meaning was paramount. Team meetings were vital in discussing cultural norms and expectations of male partners and to understand how those fed into women’s choices and preferences. Weekly meetings were held where codes were developed and, later, additional meetings were had to double-check the meaning of codes and themes, with full deference to those authors and interviewers from South Africa.

#### Interview analysis

After repeated reading of transcribed semi-structured interviews, authors CMA, CM, and JH used principles from thematic analysis to independently code interviews in MAXQDA 2022 [22]. Interview analysis sought to explore the perceived relationship strength, if relationship strength impacted a woman’s interest in male partner engagement in ANC services, and how relationship strength impacted a woman’s preferred strategy to invite her male partner into ANC. Authors CM and JH collaboratively generated 31 deductive codes from previous research about male engagement preferences and 33 inductive codes and placed them within 14 themes. The final framework had > 85% inter-rater reliability after seven meetings.

## Results

We interviewed 36 pregnant women receiving ANC services at a rural South African district hospital. Participants were a median age of 28 years (IQR: 24, 34), completed a median of 12 years of formal education (IQR: 11, 12), and 91% self-identified as Black or African (three refused to respond). Most lived with their parents/family (59%, n = 21), a third (33%, n = 12) lived with their male partners, and three lived alone (8%). Participants described themselves as single (58%, n = 21) or married (42%, n = 15). All except one participant were still in an intimate relationship with the father of their fetus.

### Current relationship quality

Most participants spoke of their relationships in positive ways. Expectations for a good relationship were focused on four components: 1) financial support to cover the costs of food, transportation, baby clothes, and/or electricity, 2) emotional support through communication, trust, and encouragement, 3) physical support through shared household chore and childcare responsibilities, and 4) a good relationship with in-laws. One woman, who spoke positively about her relationship explained why she was happy with her partner.

Pregnant Woman: Everything is very well. My mother-in-law is very good to me as well as my sisters-in-law. Also, my husband is very focused he is not the kind of person to be all over the street [drinking or cheating].

Interviewer: How does the father of your child support you in this situation?

Pregnant Woman: He takes good care of me. We are staying together in Pretoria. I only came back [to Mpumalanga] because I want to give birth at home. When I don’t feel well, he gets very worried. Even now I don’t feel okay sometimes I feel like vomiting, even when I can call and tell him the doctor said I’m not okay I know he will get worked up.

(Married, 25 years old)

Another woman reported being happy with her partner and highlighted the importance of communication and monogamy in her relationship.

We have never had serious problems, every time when we have a problem, we are able to speak about it and solve it. The relationship is good the way I see it, I am happy with him, and he is also happy, we haven’t had issues of cheating ever since we started dating.”

(Single, 23 years old)

While most women spoke positively about their relationships, a substantial minority, about 25%, reported being unhappy with their partner. Difficulties arose around finances, emotional and physical support, substance abuse, and issues with the partners extended family. One woman described the daily frustrations she has with her partner.

You have to nag, when you want him to do something, you must fight. If you don’t fight, nothing will happen. Just like this morning I wanted money for transport, he says “why didn’t you tell me yesterday”. How would I have told him? When I got back from work, he wasn’t home, since he left in the morning, and he didn’t go to work. On the other side [at the house we share] there is no electricity and he went to his home [where his children from a previous relationship live], they called him [because] there are other issues there. But since he was there until I came back from work [name redacted] and I arrive at home, I found out that electricity wasn’t fixed. On the other side we are out of gas, I am sure I have been telling him for a while and we use the gas when there is no electricity but because he went home to his kids, he didn’t refill the gas. What would I have used to cook? Yooh it is difficult.

(Single, 43 years old)

Another noted her frustration with her partners alcohol use and lack of financial support.

Pregnant woman: I was well settled where I am but now, I am no longer in a good relationship, my partner is also unemployed, I am the one buying food with grant and now I receive grant for two people.

Interviewer: Does he support you financially?

Pregnant woman: Finances no, he loves alcohol

(Married, 36 years old)

Despite frustrations, most of the participants remained with their male partners and spoke of no plans to end the relationship.

### Relationship expectations

#### Financial support

Most women reported receiving money from their partner when they specifically asked for financial assistance, whether it be for food, transportation, or items needed once the baby arrived. Nevertheless, with high rates of poverty, about half of our participants relied on more than just the partner for financial stability. One woman explained,

I would not say he supports me well financially because he does not work well…my brothers…buy use food …and also give the children money to carry to school.

(Single, 30 years old)

There were a few women in more financially stable situations. One woman was married and was expecting her first child with her partner. Her partner worked out of town and only visited one weekend a month. Financially, she indicated satisfaction with the support he provided, explaining,

I am the one handling finances and money, when his money comes in, it comes into my hands. I am happy and I don’t stress, and I don’t consult when I do something I just do what the money allows me to do.

(Married, 31 years old)

This level of financial control over family finances was unusual in our sample, only one other woman (married, aged 34 years) reported control over the money as it came in.

#### Emotional support

Even when their partners were living apart, most women reported that their emotional needs were met. Emotional needs included listening to her challenges and offering support, caring for her when she is feeling stressed, and being supportive of mood swings experienced during pregnancy. For example, one 21-year-old woman, who was expecting her second child, described her partner as supportive. She explained, “The relationship is good, we love each other, and we communicate well, we help each other to solve problems, when I tell him about my problems he assists and there is no problem” (Married, 21 years old).

#### Physical support

Physical support focused on assistance with household chores, including washing clothing, cooking food, and cleaning the house. Given that many women did not live with their male partners, physical support was only relevant for a subset of participants. Some noted that their partners were, willing to “*clean*, *wash clothes and …cook*.” (Married, 21 years old) Others noted that their partners were less inclined to physically help them but did not want to see them struggle. One woman explained, “I once complained about my back hurting and I could not bend anymore, so he bought me a washing machine to replace his laziness…” (Single, 32 years old).

### Preferences for male partner engagement in ANC and HIV testing

We asked participants if they were interested in their male partner engaging in four services: (1) ANC appointments, (2) couples HIV testing and counseling, (3) relationship counseling, and (4) the delivery. Eighty-nine percent of participants (n = 32) were interested in having their partner attend ANC services with them, 92% (n = 33) wanted couples counseling during the ANC period, 85% (n = 28), excluding those who reported already knowing their partner’s status and disclosing their own, wanted couples HIV testing and counseling, and 81% (n = 29) wanted her partner to attend the birth of their child. Women believed that having her male partner present during clinical visits would provide an opportunity to complete couple-based HIV testing and counseling, which would improve trust and provide the partner an understanding of what happens during pregnancy and give him the opportunity to better understand her struggles during pregnancy. Among women who wanted their partner to participate in ANC services, 50% (n = 18) preferred to verbally invite their partner without assistance, 41% (n = 15) preferred an invitation letter from the health facility, and 27% (n = 10) preferred a trained male CHW undertaking counseling with her partner before that CHW issued an invitation (some women indicated a preference for more than one intervention).

**Fig 1:**
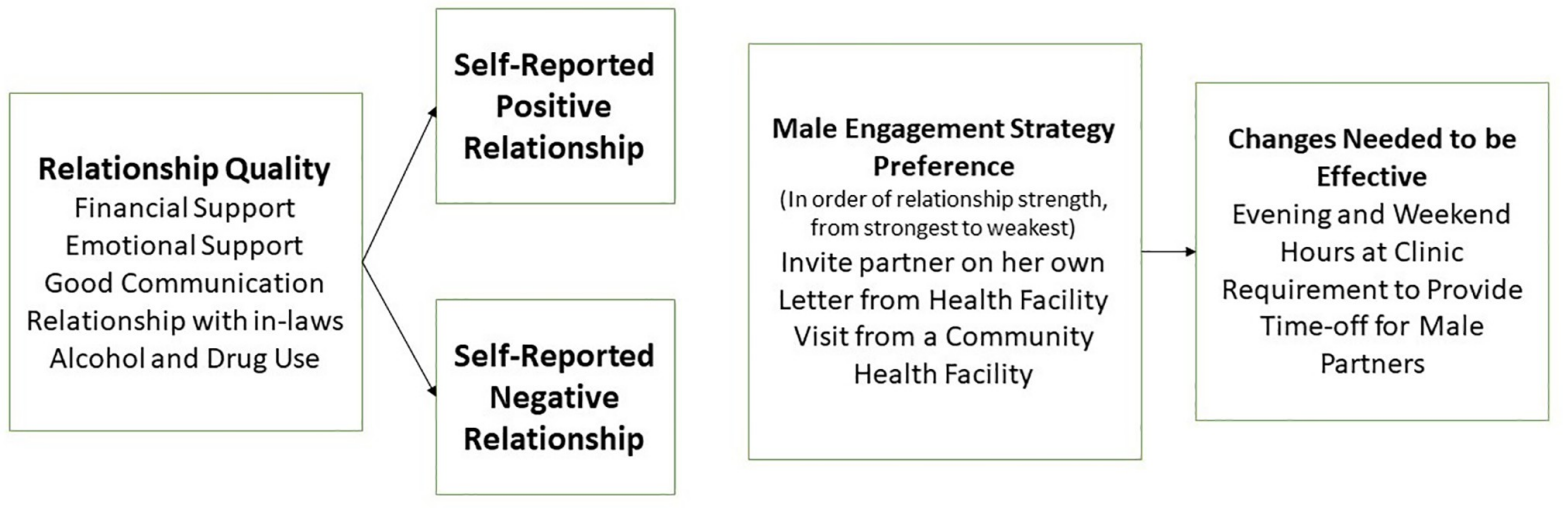
Association of relationship strength to male partner engagement strategy preference.

The selection of a particular engagement strategy tended to reflect the strength of a couple’s relationship (Fig 1). Those who reported a positive relationship with their partner typically preferred inviting him to attend ANC services without the assistance of the health system. One woman explained, “I prefer number 2 [verbal invite] because when I talk to him, he listens” (Married, 31 years old). Another woman stated,

When there is communication there will not be a need for a community health worker or for a letter. When he does not understand [will not attend ANC services] then you [the health facility] can write him a letter or send a community health worker.

(Married, 27 years old)

Pregnant women in unhappy, difficult relationships reported that if they extended the invitation to attend ANC visits, her partner may not attend clinic. A letter from the health facility, however, was seen as an official invitation that could not be easily ignored. A pregnant woman who reported being in an unhappy relationship with her partner indicated that she preferred an invitation letter “so that he can believe” [he should attend clinic with her] (Single, 35 years old). Another reported that “a letter can be very helpful, so if he does not show up, then he can come and explain himself why he did not honor the letter.” (Single, 42 years old).

Factors other than relationship strength also played a role for some women who preferred the invitation letter. For example, several participants noted that South African cultural norms do not encourage for men to take time off work to attend ANC services. One woman elaborated that a letter from the health facility would provide her husband written documentation to show to his employer. She explained, “I would prefer that you write a letter, the problem that my husband has is that he works at the farms, and they would need proof that it is a matter of clinic…” (Married, 21 years old).

Women not as comfortable speaking directly with their partner expressed a preference for having a CHW invite their partner to ANC. Three situations emerged where women preferred a CHW-initiated partner invitation: a partner with a history of violence, a partner who had abandoned her, or a new relationship where the couple have not yet established a strong relationship. One woman described her tumultuous relationship:

Things are difficult I don’t want to lie, he left me when I was 2 months pregnant. I had no money to come here I had to try somewhere. On the side I buy baby clothes, I just live a trying life. Nothing is going well, I am suffering. … you can send a community health worker to go and talk to him.

(Single, 25 years old)

Women in this situation hoped that the CHW could convince her partner to provide some type of support, or at least to get him to the health facility where health care workers would provide guidance on how to support her during the pregnancy.

### Will a partner attend an ANC appointment? A woman’s perspective

Most women believed their partners would attend her ANC appointment if invited; however, financial and clinical structural barriers could limit male partner participation, even among interested partners. For example, given that clinical appointments are offered during typical working hours, local employers would have to allow men to miss work to attend appointments. One woman explained,

I like them [the ideas of male partner participation in ANC and birth] but most of what you said like coming with me to [ANC] checkups, will be an issue because of the distance and he only gets paid when he clocks in, although he keeps saying that he is doing it for the child and his off days are usually on weekends and does not correspond with my hospital appointments

(Single, 20 years old)

While we did not specifically ask women where their partner worked, several women revealed that their partners worked in mining or farming industries some distance from both the hospital and their homes which resulted in the male partner only returning home once per month. Despite potential interest, the partner would likely not be able to attend ANC appointments unless clinical service times were adapted.

Others highlighted the challenges they experienced being the newest partner in polygamous relationships. The first wife was perceived as having control over the man’s behavior, reducing the likelihood of his participation in ANC services for the wife being interviewed. One woman explained,

It would not be possible because I am not always with him. The truth is that he supports me with everything, but I am his second wife, and we [do] get along with the sister wife, [but] there are fights. Before I became pregnant, he informed his wife there is another wife… I don’t have my own house and I still live at home, sometimes he gets to be with me for a few days; sometimes we go to his home. If it was up to me, I would say I am interested [in him attending ANC appointments] but I can’t be able to be with him when I am supposed to be…he won’t have the time for them to be with me.

(Single, 28 years old)

## Discussion

Most of the interviewed pregnant women would like to engage their male partners in ANC services, even when their relationships are, in their own estimation, unsatisfactory. Issues that drove women to report frustrations with their partners included poor communication, a lack of financial or emotional support, polygamy, and misuse of alcohol. These issues reflect the results of similar studies among couples in South Africa [20, 33–35]. Women who report difficult relationships may benefit the most from male partner engagement in ANC. Couples with good communication skills may need little encouragement to test together or discuss their HIV status. Disclosure is correlated with improved health outcomes, condom use, and good relationship with an intimate partner [36, 37]. Facilitating couple-based HIV testing and counseling in a supportive environment, particularly among those experiencing relationship challenges, would like lead to increased disclosure–and support- within intimate partner relationships [27, 38]. It also may also reduce the risk of intimate partner violence (IPV) [39, 40].

More than 80% of interviewed pregnant women wanted their partners to attend ANC appointments, complete couples HIV testing and counseling together, and provide support during the birth of their child. This support presents the health system with an opportunity to develop a partner engagement protocol, one where women can opt their male partner into participation in ANC, delivery, and post-natal care. Evidence-based couple-engagement programs that reflect the requests of our study population have been successfully implemented as clinical trials in South Africa, leading to increased uptake of male partner participation [26]. The remaining challenge is integration of these programs into the national health system.

Given the overwhelming interest in partner engagement, clinical practices will need to adjust. Effective implementation will take an investment in infrastructure, to ensure privacy for all women receiving ANC services or in active labor and will require additional staff training to provide effective based care. For women with migrant worker partners, clinical hours may need to be offered in the evenings or weekends to accommodate their needs. Programs expanding services to allow for men to attend clinic after hours have successfully implemented in Kenya and South Africa [41–43]. For women with self-reported difficult relationships, a counselor may need to be available to guide discussions, including around partner support and HIV testing and counseling.

Unsatisfactory relationships are likely to be put under increased pressure during the pregnancy and post-partum period in South Africa and around the world [15, 33], leaving women with few avenues to secure their partners emotional and financial support. With 49% of the population living on less than 1,183 Rand (USD 70.90 in 2015) per month [44], women are particularly vulnerable during periods when employment is interrupted. Worldwide, women make only 77 cents for every dollar earned by men, and this inequity is exacerbated by restrictive parental leave which can push women into part-time or informal employment [45]. In South Africa, much like in other parts of the world, maternity leave support is limited or non-existent, particularly in the informal employment sector. The need for support may lead some women to stay in unsatisfactory relationships, even if the benefits are limited and the risks are high. Pregnant women around the world would benefit if they had access to psychosocial support during ANC, including relationship counseling, to help them make difficult decisions during a time already characterized by change.

## Conclusions

Rural South African women, even those in unsatisfactory relationships, want their male partners to attend their ANC visits and delivery. To make this possible, health facilities need to contextualize male partner engagement outreach strategies to the preferences and needs of the pregnant woman. Future studies are required to assess how personalized care to pregnant women can be delivered most cost-effectively to ensure they receive the support they need. To further partner involvement in clinical care, additional trainings in couples HIV testing and counseling for nurses as well as expanding and updating health facilities to accommodate partners in appointment rooms will likely be necessary.

## Supporting information

S1 File Inclusivity in global research. (DOCX)
